# Synthesis of Polyfluorinated Thia- and Oxathiacalixarenes Based on Perfluoro-*m*-xylene

**DOI:** 10.3390/molecules26030526

**Published:** 2021-01-20

**Authors:** Vladimir N. Kovtonyuk, Yuri V. Gatilov, Pavel V. Nikul’shin, Roman A. Bredikhin

**Affiliations:** N.N. Vorozhtsov Novosibirsk Institute of Organic Chemistry, SB RAS, 9 Academician Lavrentjev Ave., 630090 Novosibirsk, Russia; gatilov@nioch.nsc.ru (Y.V.G.); npv@nioch.nsc.ru (P.V.N.); bredikhin_ra@mail.ru (R.A.B.)

**Keywords:** perfluorinated tetrathiacalix[4]arene, perfluoro-*m*-xylene, dioxadithiacalix[4]arenes, tetraoxadithiacalix[6]arene, thiourea, 2,5-difluoro-4,6-bis(trifluoromethyl)benzene-1,3-dithiol, X-ray analyses.

## Abstract

Perfluorinated tetrathiacalix[4]arene was obtained by heating perfluoro-*m*-xylene with thiourea or 2,5-difluoro-4,6-bis(trifluoromethyl)benzene-1,3-dithiol at 90 °C. Interaction of perfluoro-*m*-xylene with resorcinol or orcinol under mild conditions and subsequent heating of the mixture with 2,5-difluoro-4,6-bis(trifluoromethyl)benzene-1,3-dithiol leads to polyfluorinated dioxadithiacalix[4]arenes. Triphenyl and pentaphenyl ethers formed by the interaction of perfluoro-*m*-xylene with resorcinol under heating with thiourea gives polyfluorinated oxathiacalixarenes containing six and five aromatic nuclei, respectively.

## 1. Introduction

Thiacalixarenes are one of the widely used platforms in supramolecular chemistry; this is due to their unique ability for complex formation with various metal cations [1,2]. Thiacalixarenes are used to create sensors [1,3,4], catalysts [5,6], molecular magnets [7], and luminescent materials [1,8]. This widespread usage of thiacalixarenes is due to a fairly simple synthesis method based on the interaction of phenols with sulfur in the presence of NaOH [1,9]. Other synthesis methods are based on the aromatic nucleophilic substitution reactions in 1,3-dihalogenarenes or hetarenes [10,11,12,13,14,15]. Sodium sulphide [10] or sodium hydrosulphide [11], plus dithioresorcinol [12,13,14,15], can be used as the S-nucleophile in these reactions, which leads to the formation of thiacalixarenes containing several (*n* ≥ 3) identical aromatic (heteroaromatic) rings, as well as macrocycles with alternating acceptor and donor arenes.

We have previously shown that the interaction of polyfluoroaromatic compounds (perfluoro-*m*-xylene, pentafluorobenzonitrile and pentafluoronitrobenzene) with various resorcins and bisphenols leads to the formation of polyfluorinated tetraoxacalixarenes with a good yield [16,17,18,19]. Interest in fluorinated tetraoxacalixarenes is associated with a fairly high electron-deficiency of polyfluorinated aromatic nuclei in these compounds, which may increase their ability with regard to host–guest intermolecular interactions.

In this paper, the possibility of polyfluorinated thia- and oxathiacalixarenes synthesis based on the reactions of perfluoro-m-xylene with thiourea and 2,5-difluoro-4,6-bis(trifluoromethyl)benzene-1,3-dithiol was investigated. This is of interest for studying the possibility of the polyfluorinated thia- and oxathiacalixarenes complexation with various metal cations, since the bridged sulfur atoms in thiacalixarenes can directly coordinate with metal ions [1].

## 2. Results and Discussion

**Scheme 1:**
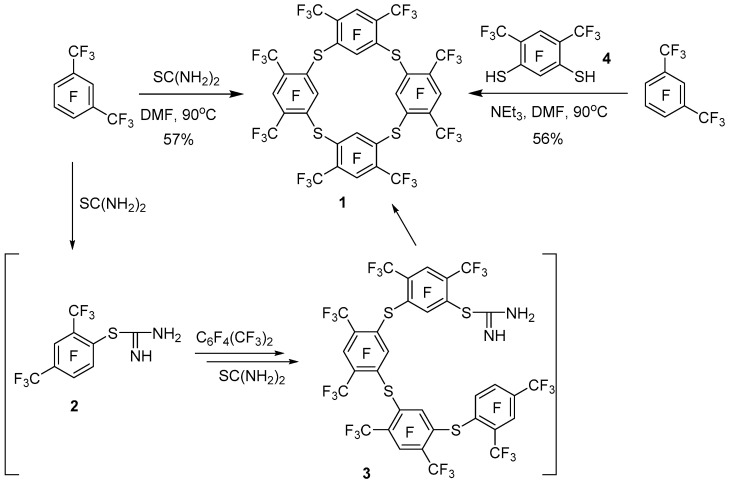
Formation of perfluorinated tetrathiacalix[4]arene **1.**

Earlier in the work of Tatlow [20], it has been shown that the interaction of polyfluorinated aromatic compounds with thiourea, under mild conditions, is a convenient method for the synthesis of diaryl sulphides, but in the case of perfluoro-*m*-xylene, polymerization of the latter was observed. It should be noted that the composition of the oligomeric mixture in this reaction has not been studied. It may be assumed that linear oligomers with a low polymerization depth will form predominantly in this reaction under mild conditions with a sufficiently high concentration of the initial perfluoro-*m*-xylene. We performed this reaction under conditions favored by cyclo-oligomerization. Indeed, when a mixture of perfluoro-*m*-xylene with an excess of thiourea is heated at 80–90 °C in a diluted (c~0.08 mol/L) DMF solution, perfluorinated tetrathiacalix[4]arene **1** is formed as the main product (Scheme 1).

It is assumed that the interaction of polyfluoroaromatic compounds with thiourea proceeds through the intermediate formation of an isothiouronium derivative of type **2**, which then acts as an ArS^−^ equivalent in the reaction with polyfluoroarene, giving diaryl sulphides or linear oligomers as the products [20,21]. Subsequent oligomerization in the case of perfluoro-*m*-xylene leads to the intermediate formation of isothiouronium **3** macrocyclization of which gives tetrathiacalixarene **1** (Scheme 1).

**Scheme 2:**
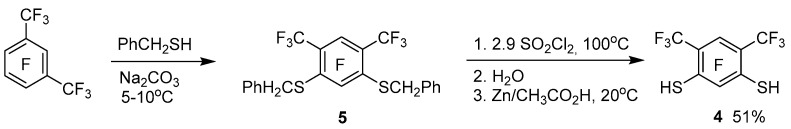
Synthesis of dithiol **4.**

We also obtained tetrathiacalix[4]arene **1** by interaction of perfluoro-*m*-xylene with dithiol **4** (Scheme 1). The latter was synthesized by us from perfluoro-*m*-xylene via intermediate formation of bis(benzylthio)benzene **5** according to Scheme 2. The standard deprotection method [22] in compound **5**, due to the presence of acceptor substituents, does not lead to the formation of dithiol **4.** Therefore, based on studies of the reactivity of polyfluorinated arenthiols [23], for deprotection of the thiobenzyl group in compound **5**, it was proposed to use chlorination by SO_2_Cl_2_, hydrolysis and subsequent reduction by Zn of the resulting product mixture.

**Scheme 3:**
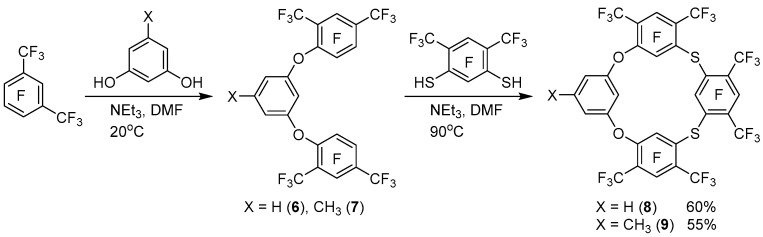
Synthesis of dioxadithiacalix[4]arenes **8** and **9.**

We have previously shown that the reaction of perfluoro-*m*-xylene with resorcinol under mild conditions led to the formation of a mixture of polyphenyl ethers with a predominant content of triphenyl ether [17]. Further heating of this mixture with resorcinol or tetrafluororesorcinol gave polyfluorinated oxacalixarenes of the ABAB or ABAC type. The same approach was used in the reactions of pentafluorobenzonitrile and pentafluoronitrobenzene with various resorcinoles [18,19], and the synthesis was also performed without intermediate isolation of triphenyl ethers. We used this approach for the synthesis of dioxadithiacalix[4]arenes. Thus, the interaction of two equivalents of perfluoro-*m*-xylene with the equivalent of resorcinol or orcinol under mild conditions and subsequent heating of the reaction mixtures with the equivalent of dithioresorcinol **4** leads to the formation of polyfluorinated dioxadithiacalix[4]arenes **8, 9** with a good yield (Scheme 3).

**Scheme 4:**
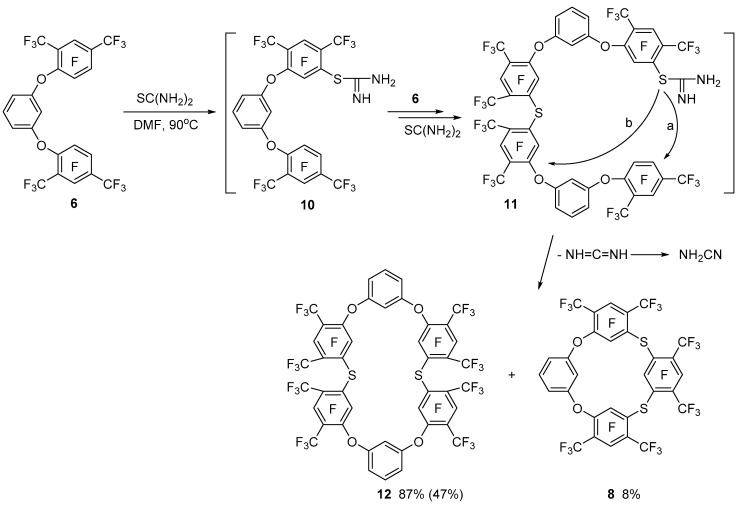
Interaction of 1,3-bis[3,5,6-trifluoro-2,4-bis(trifluoromethyl)phenoxy]benzene **6** with thiourea.

At the same time, the interaction of triphenyl ether **6** with thiourea leads to tetraoxadithiacalix[6]arene **12** and dioxadithiacalix[4]arene **8** as the main and minor products, respectively (Scheme 4). The intermediate isothiouronium derivative **10** formed in this reaction then reacts successively with another equivalent of triphenyl ether **6** and thiourea giving another isothiouronium derivative **11.** Macrocyclization of derivative **11** can take place both on the terminal (main pathway a) and the internal (minor pathway b) perfluoro-*m*-xylene fragments to form tetraoxadithiacalix[6]arene **12** and dioxadithiacalix[4]arene **8**, respectively. Intramolecular cyclization of isothiouronium derivative **10** with the formation of dioxatiacalix[3]arene is unlikely, which can be explained in terms of strain of the intended cycle.

**Scheme 5:**
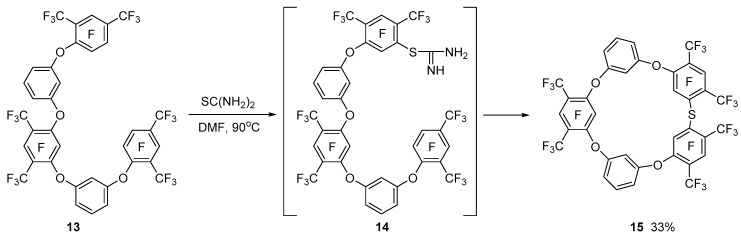
Formation of tetraoxathiacalix[5]arene **15.**

In contrast, the reaction of pentaphenyl ether **13** with thiourea intramolecular macrocyclization of isothiouronium derivative **14** leads to the formation of tetraoxatiacalix[5]arene **15**, which is due to a decrease in transannular strain in the cycle (Scheme 5).

The structure of thia- and oxathiacalixarenes **1, 8, 9, 12, 15** was determined based on analytical and ^1^H, ^19^F, ^13^C NMR data (Supplementary Material Figures S1_F–S13_C). The structure of tetrathiacalixarene **1** was also confirmed by X-ray data.

**Figure 1:**
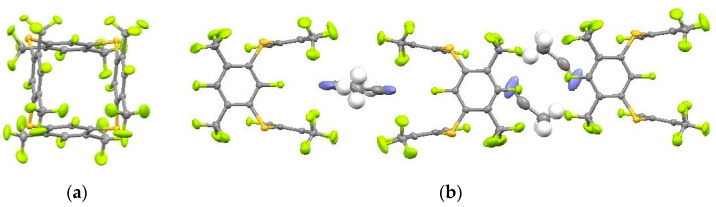
Molecular structure of tetrathiacalixarene **1** (**a**) and complex 1*2CH_3_CN (**b**).

According to X-ray analysis of the single crystal obtained from CH_2_Cl_2_, tetrathiacalixarene **1** is in the 1,3-alternate conformation, which is typical for tetrathiacalixarenes that do not have substituents in the inner cycle [13,15,24] (Figure 1a). The sulphur atoms are located in the same plane practically without deviation. The C–S bond length 1.78 Å corresponds to the literature data for tetrathiacalixarenes [15,24]. The C–S–C angles are 100.6–100.7°, and the torsion angles around the C–S bonds are 57.8–60.3°. The sulphur atoms are slightly displaced outside from the planes of the aromatic nucleus, the deviation is 0.12–0.21 Å. The opposite aromatic nuclei are located almost parallel to each other, and the dihedral angles are 2.44° and 4.69°, respectively. It should be noted that the difference in the dihedral angles for the tetrathiacalixarenes described in the literature is significantly higher (2–130°) [15,24]. Crystallization of tetrathiacalixarene **1** from acetone or acetonitrile leads to the formation of complexes including 1 or 2 solvent molecules (Figure 1b). In this case, the dihedral angles between the opposite aromatic nuclei increase to 19.2° and 20.1° (1*2CH_3_CN).

One set of three signals in the ^19^F NMR spectra (Supplementary Material Figure S3_F) of tetrathiacalixarene **1** without significant broadening in the signal structure at room temperature can indicate both the realization of one symmetric conformation and a very fast conformational interconversion in the NMR time scale. It can be assumed that the presence of fairly large eight-CF_3_ groups in the tetrathiacalixarene **1** molecule should shift the equilibrium towards the least sterically hindered 1,3-alternate conformation similar to that determined by the X-ray method for the crystal state. A similar 1,3-alternate conformation was proposed earlier in the analysis of ^1^H NMR spectra for solutions of tetrathiacalixarene without substituents in the internal macrocycle [14]. When four volume substituents (OC_2_H_5_) are introduced, the interconversion becomes difficult, and the equilibrium between all four possible conformations of tetrathiacalixarene according to NMR spectra is fixed [25].

We have previously observed a noticeable upfield shift of the inner-rim hydrogen and fluorine atoms of the resorcinol fragments in the ^1^H and ^19^F NMR spectra of polyfluorinated tetraoxacalixarenes, which is characteristic for this class of compounds [16,17,18,19]. The value of this upfield shift is solvent dependent, which may be due to implementation of some equilibrium 1,3-alternate conformations characterized by different degrees of magnetic shielding of the inner-rim protons and fluorines of the resorcinol fragments by the neighboring aromatic rings [19].

**Figure 2:**
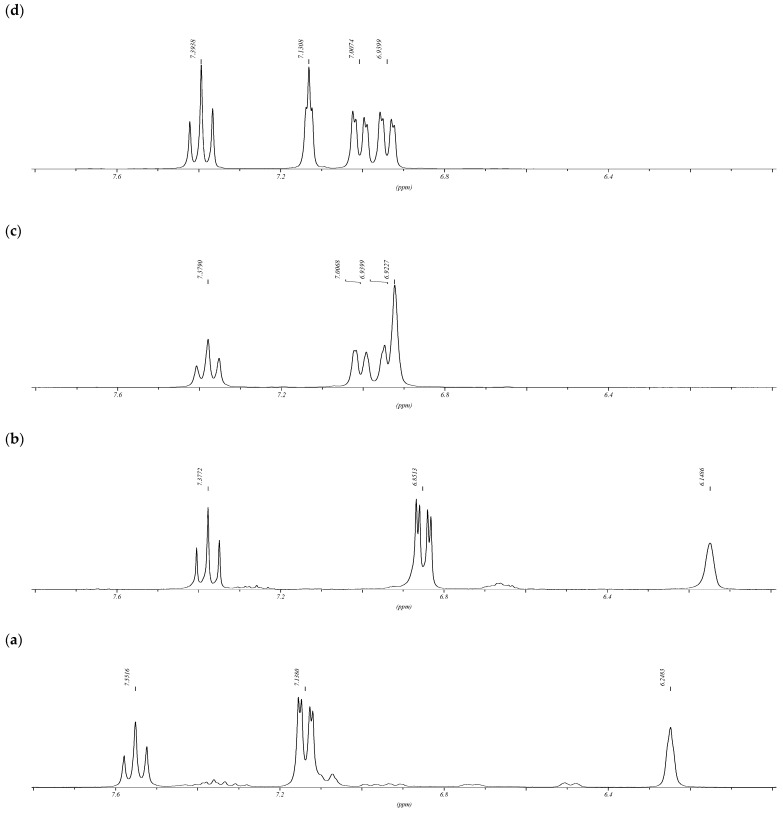
^1^H NMR spectra (acetone-d6) of dioxadithiacalix[4]arenes **8** (**a**), tetraoxadithiacalix[6]arene **12** (**b**), tetraoxathiacalixarene **15** (**c**), pentaphenyl ether **13** (**d**).

In the ^1^H NMR spectra of dioxadithiacalix[4]arenes **8–9,** an upfield shift of the inner-rim protons (H-28) of the resorcinol fragment is also observed, and the value of this shift depends on the solvent (Supplementary Material Figure S5_H). So, the chemical shift of the hydrogen atom H-28 in CDCl_3_ is 5.45 ppm for **8** and 5.08 ppm for **9**, and in (CD_3_)_2_CO 6.25 ppm for **8** (Figure 2a) and 6.00 ppm for **9**. The presence of an upfield shift of the inner-rim protons in the ^1^H NMR spectra indicates that dioxadithiacalix[4]arenes **8**–**9**, as well as tetraoxacalixarenes [26], have a 1,3-alternate conformation in solution. In the ^1^H NMR spectra of tetraoxadithiacalix[6]arene **12**, a noticeable upfield shift of the inner-rim protons (H-39,42; δ 6.14 ppm in acetone-d_6_, Figure 2b) of the resorcinol fragment is also observed, and it is practically absent in the ^1^H NMR spectra of tetraoxathiacalixarene **15** (H-33,35; δ 6.92 ppm in acetone-d_6_, Figure 2c). For comparison, the chemical shift of the hydrogen atom, located between two perfluorinated phenoxy fragments, in triphenyl ether **6** (H-2; δ 7.10 ppm in acetone-d_6_) and pentaphenyl ether **13** (H-2′; δ 7.13 ppm in acetone-d_6_, Figure 2d) can be used as reference points. It should be noted that, in contrast to tetraoxadithiacalix[6]arene **12**, the ^1^H NMR spectra of closely related polyfluorinated hexaoxacalix[6]arenes are lacking for an upfield shift of the inner-rim hydrogen atoms signals [17].

## 3. Materials and Methods

### 3.1. General Methods

Thiourea, resorcinol and orcinol monohydrate were obtained from Aldrich (Milwaukee, WI, USA), AppliChem (Darmstadt, Germany) and FluoroChem (Hadfield, UK), respectively, and used directly without further purification. Dimethylformamide and triethylamine were held over NaOH during a week and distilled immediately before use. Perfluoro-*m*-xylene was a ~3:1 mixture with its *para-*isomer (according to the ^19^F NMR data) which is considerably less reactive [27]. This mixture is a byproduct in the synthesis of octafluorotoluene by interaction of hexafluorobenzene with Teflon chips [28,29].

The ^19^F and ^1^H NMR spectra were recorded for solutions in CDCl_3_ and OC(CD_3_)_2_ on a Bruker AV300 spectrometer at 282.36 MHz and 300 MHz, respectively, ^13^C NMR spectra were recorded on a Bruker AV-400 instrument (100.6 MHz). Chemical shifts are given in δ ppm from CCl_3_F (^19^F) and TMS (^1^H, ^13^C), J values in Hz; C_6_F_6_ (−162.9 ppm from CCl_3_F), CHCl_3_ (7.24 ppm from TMS), OC(CD_3_)_2_ (2.05 ppm from TMS), OC(CD_3_)_2_ (29.8 ppm from TMS) were used as internal standards.

The X-ray diffraction experiments for **1** and 1*2CH_3_CN were carried out on a Bruker KAPPA APEX II diffractometer with graphite monochromated MoKα (*λ* = 0.71073 Å) radiation at 296 K. Experimental data reduction was performed using APEX2 suite [30]. The structures were solved by direct methods and refined by the full-matrix least-squares technique against F^2^ in the anisotropic-isotropic approximation. The H atom positions in 1*2CH_3_CN were calculated with the riding model. All calculations were performed using SHELXL-2018/3 [31]. All CF_3_ groups in crystal **1** are disordered over two positions with SOF 0.21–0.74 and some restrictions were applied.

Gas chromatographic–mass spectrometric analysis (GC–MS) was performed on a Hewlett Packard HP 5890 Series II chromatograph coupled with an HP 5971 mass-selective detector (HP-5MS capillary column, 30 m × 0.25 mm, film thickness 0.25 μm; carrier gas helium, flow rate 1 mL/min; oven temperature programming from 50 °C (2 min) to 280 °C at a rate of 10 °/min and finally 5 min at 280 °C; injector temperature 280 °C; ion source temperature 175 °C; electron impact, 70 eV; 1.2 scan/s, a.m.u. range 30–650). HPLC analysis was performed on a Milichrome-A-02 (Econova, Novosibirsk, Russia). Column—2.0 × 75 mm with ProntoSIL-120-5-C18 (BISCHOFF Analysentechnik U.—GERÄTE GMBH, Leonberg, Germany). Temperature of column 30 °C. Eluent: methanole/water, gradient from 4:1 to pure methanole (1500 mcl) and then methanole (1500 mcl). The reference wavelength—260 nm.

The elemental compositions of thiacalixarene **1** and oxathiacalixarenes **8, 9, 12, 15,** were determined by classical methods and their molecular weights were determined in acetone solution at 40 °C using a Knauer K-7000 osmometer. The elemental compositions of compounds **4, 5,** were determined from the high-resolution mass spectra which were recorded on a Thermo Scientific DFS instrument (electron impact, 70 eV).

The progress of reactions was monitored by TLC on Silica gel 60 F254 plates (Merck, Darmstadt, Germany). Silica gel (0.063–0.200 mm; Merck, Darmstadt, Germany) was used for column chromatography.

### 3.2. Synthesis of 2,5-difluoro-4,6-bis(trifluoromethyl)benzene-1,3-dithiol ***4***

#### 3.2.1. 1,3-Bis(benzylthio)-2,5-difluoro-4,6-bis(trifluoromethyl)benzene **5**

Na_2_CO_3_ (3.11 g, 10.9 mmol) and 18.82 g (58.3 mmol) benzyl mercaptan was added successively to a stirred solution of 10.04 g of a mixture of perfluorinated *m*- and *p*-xylenes (~26.3 mmol of the *meta-*isomer) in 80 mL of dimethylformamide. The solution was stirred for 18 h at 5–10 °C, and then poured into 160 mL of 10% aqueous HCl solution. The precipitate was filtered out and dried over CaCl_2_. Solid product (12.51 g) contained 85% of dithiol **4**, according to HPLC data. Analytical sample was obtained by crystallization from hexane. Mp 62.3–63.7 °C. ^19^F NMR (CDCl_3_): δ −116.0 (septet d, 1F, *J* = 36 Hz, *J* = 15 Hz, F-5), −93.6 (d, 1F, *J* = 15 Hz, F-2), −55.5 (d, 6F, *J* = 36 Hz, 2CF_3_). ^1^H NMR (CDCl_3_): δ 4.10 (s, 2H, CH_2_), 7.15–7.28 (m, 5H, C_6_H_5_). Anal. Calcd for C_22_H_14_F_8_S_2_: C 53.44; H 2.85; F 30.74; S 12.97%. Found: C 53.85; H 2.79; F 31.18; S 12.80. HRMS *m/z*: 494.0403 (M^+^). Calculated: M = 494.0404.

#### 3.2.2. 2,5-Difluoro-4,6-bis(trifluoromethyl)benzene-1,3-dithiol **4**

Compound **5** (11.12 g, 19.1 mmol, technical product with an 85% content) and SO_2_Cl_2_ (7.52 g, 55.7 mmol) were placed in a glass ampoule. The ampoule was sealed and heated in a metal casing for 16 h at 100–110 °C. The reaction mixture was cooled and poured into 200 mL of water, then 10 mL EtOAc, 40 mL CHCl_3_ and 9.41 g (88.8 mmol) Na_2_SO_3_ were added. After stirring, the organic layer was washed with water (2 × 200 mL) and the solvents were evaporated. The oily product was dissolved in 70 mL of glacial acetic acid, and then Zn (12.62 g, 192.9 mmol) was added by small portions and stirred for 24 h at 20 °C. The reaction mixture was treated by 60 mL of 10% aqueous HCl solution, and the product was extracted with CHCl_3_ (3 × 25mL) and additionally with EtOAc (3 × 15 mL). The solvents were evaporated, and the product was distilled with steam. Two fractions were obtained: solid (3.51 g) and liquid (1.18 g) with a dithiol **4** content of 87% and 52%, respectively (GC–MS). Pure dithiol **4** (1.86 g, 51% with 98% content according to GC–MS) was obtained by crystallization of solid fraction from hexane. Mp 66.4–67.2 °C. ^19^F NMR (CDCl_3_): δ −115.7 (septet d, 1F, *J* = 27 Hz, *J* = 15 Hz, F-5), −103.6 (m, 1F, F-2), −57.1 (dd, 6F, *J* = 27 Hz, *J* = 8 Hz, 2CF_3_). ^1^H NMR (CDCl_3_): δ 4.36 (quintet, 2H, *J* = 9.7 Hz, SH). ^13^C NMR (CDCl_3_): δ 113.5 (qdd, ^2^*J_CF_* = 33.6 Hz, ^2^*J_CF_* = 15.8 Hz, *J_CF_* = 1.7 Hz, C–CF_3_), 122.2 (qdd, *^1^J_CF_*= 275.6 Hz, *J_CF_* = 2.3 Hz, *J_CF_* = 1.5 Hz, CF_3_), 127.8 (dd, *^2^J_CF_* = 28.1 Hz, *J_CF_* = 3.2 Hz C-SH), 147.9 (dd, *^1^J_CF_*= 231.0 Hz, *J_CF_* = 3.7 Hz C-F), 155.4 (dm, *^1^J_CF_*= 266.9 Hz, C–F). Anal. Calcd for C_8_H_2_F_8_S_2_: C 30.58; H 0.64; F 48.37; S 20.41%. Found: C 30.16; H 0.51; F 48.33; S 20.05. HRMS *m/z*: 313.9472 (M^+^). Calculated M = 313.9465.

### 3.3. Synthesis of Polyfluorinated Triphenyl and Pentaphenyl Ethers ***6***, ***13***

Solution of trimethylamine (1.60 g, 16 mmol) and resorcinol (0.36 g, 3.3 mmol) in 10 mL of acetone was added dropwise to a stirred solution of 2.20 g of a mixture of perfluorinated *m*- and *p*-xylenes (~6 mmol of the *meta-*isomer) in 15 mL of acetone. The solution was stirred for 20 h at 20 °C, and solvent was evaporated. Column chromatography (eluents petroleum ether (40–70 °C)/CCl_4_ 1:2, 1:3, 1:5) on silica gel gave 1.16 g (1.8 mmol, 60%) of triphenyl ether **6,** 0.45 g (0.45 mmol, 27%) of pentaphenyl ether **13** and 0.17 g of polyphenyl ethers.

#### 3.3.1. 1,3-Bis[3,5,6-trifluoro-2,4-bis(trifluoromethyl)phenoxy]benzene **6**:

Viscous oil ^19^F NMR (OC(CD_3_)_2_): δ −149.8 (dd, 2F, F-6′), −126.6 (qd, 2F, F-5′), −115.7 (m, 2F, F-3′), −55.4 (t, 6F, 2CF_3_), −55.3 (d, 6F, 2CF_3_). ^1^H NMR (OC(CD_3_)_2_): δ 6.97 (dd, 2H, H-4,6), 7.10 (t, H, H-2), 7.41 (t, H, H-5). GC–MS, *m/z*: 642 (M^+^).

The ^1^H and ^19^F NMR spectra and the mass spectrum of triphenyl ether **6** coincide with the product data obtained from [17].

#### 3.3.2. 1,3-Bis{3-[3,5,6-trifluoro-2,4-bis(trifluoromethyl)phenoxy]phenoxy}-2,5-difluoro-4,6-bis(trifluoromethyl)benzene **13**:

Viscous oil ^19^F NMR (OC(CD_3_)_2_): δ −149.6 (dd, 2F, *J* = 20 Hz, *J* = 11 Hz, F-6′), −138.9 (d, 1F, *J* = 12 Hz, F-2), −126.6 (qd, 2F, *J* = 23 Hz, *J* = 20 Hz, F-5′), −115.6 (qqd, 2F, *J* = 28 Hz, *J* = 23 Hz, *J* = 11 Hz, F-3′), −115.2 (septet d, 1F, *J* = 28 Hz, *J* = 12 Hz, F-5), −55.5 (t, 6F, *J* = 23 Hz, 2CF_3_), −55.4 (d, 6F, *J* = 28 Hz, 2CF_3_), −55.3 (d, 6F, *J* = 28 Hz, 2CF_3_). ^1^H NMR (OC(CD_3_)_2_): δ 6.94 (dd, 2H, *J* = 8.2 Hz, *J* = 2.1 Hz, H-4′), 7.01 (dd, 2H, *J* = 8.4 Hz, *J* = 2.1 Hz, H-6′), 7.13 (t, 2H, *J* = 2.1 Hz, H-2′), 7.39 (t, 2H, *J* = 8.3 Hz, H-5′). GC–MS, *m/z*: 499 (M^++^, M 998).

### 3.4. Synthesis of Tetrathiacalixarene ***1***

Method A: Thiourea (0.60 g, 8 mmol) was added to a stirred solution of 1.60 g of a mixture of perfluorinated *m*- and *p*-xylenes (~4.1 mmol of the *meta-*isomer) in 50 mL of dimethylformamide. The solution was stirred for 3 h at 20 °C and 5 h at 90 °C, and then the solvent was evaporated in vacuum. By column chromatography on silica gel using mixture (3:1) CCl_4_ and CHCl_3_ as eluent 1.20 g of the solid product was isolated, double-play crystallization of which from CCl_4_ gave 0.64 g of tetrathiacalixarene **1.**

Method B: Triethylamine (0.80 g, 8 mmol) was added to a stirred solution of 0.35 g (1.1 mmol) of dithioresorcinol **4** and 0.80 g of a mixture of perfluorinated *m*- and *p*-xylenes (~2 mmol of the *meta-*isomer) in 70 mL of dimethylformamide. The mixture was stirred for 17 h at room temperature and 30 min at 90 °C then 0.30 g (1 mmol) of dithioresorcinol **4** was introduced and stirred for 20 h at 90 °C. The mixture after evaporation of solvent was treated with 80 mL of ~5% aqueous HCl and then with methylene chloride (3 × 50 mL). The extract was dried over Na_2_SO_4_, and evaporation gave 2.30 g of a viscous material. By column chromatography on silica gel using CCl_4_ and CHCl_3_ as eluents, 0.68 g of the solid product containing (GC–MS) 93% tetrathiacalixarene **1** was isolated.

5,11,17,23,25,26,27,28-Octafluoro-4,6,10,12,16,18,22,24-octakis(trifluoromethyl)-2,8,14,20-tetrathiapentacyclo[19.3.1.1^3,7^.1^9,13^.1^15,19^]octacosa-1(25),3(28),4,6,9(27),10,12,15(26),16,18,21,23-dodecaene **1**:

White solid (0.64 g, 57%). mp 304.6–304.9 °C. ^19^F NMR ((CD_3_)_2_CO): δ −112.1 (septet d, 4F, J_F(5)-2CF3(4,6)_ = 33 Hz, J_F(5)-F(28)_ = 15 Hz, F-5,11,17,23), −91.6 (m, 4F, F-25,26,27,28), −53.5 (dd, 24F, J_CF3(4)-F(5)_ = 33 Hz, J_CF3(4)-F(28)_ = 7 Hz, CF_3_(4,6,10,12,16,18,22,24)). ^13^C NMR ((CD_3_)_2_CO): δ 122.3 (qd, ^2^J_CF_ = 32.5 Hz, ^2^J_CF_ = 14.0 Hz, C–CF_3_), 122.5 (q, ^1^J_CF_ = 276.0 Hz, CF_3_), 127.7 (d, ^2^J_CF_ = 25.9 Hz, C–S), 155.3 (d, ^1^J_CF_ = 269.8 Hz, C–F), 158.5 (dd, ^1^J_CF_ = 244.2 Hz, J_CF_ = 3.0 Hz, C–F). Anal. Calcd for C_32_F_32_S_4_: C, 34.30; F, 54.25; S, 11.44%; M 1120. Found: C, 34.00; F, 54.37; S, 11.48%; M 1120.

Crystal data of **1**: C_32_F_32_S_4_, M = 1120.56, monoclinic, space group *P*2_1_/*c*, *a* = 12.5563(14), *b* = 15.9119(18), *c* = 18.7987(18) Å, β = 96.635(4)°, *V* = 3730.7(7) Å^3^, *Z* = 4, d_calc_ = 1.995 g·cm^−3^, μ = 0.444 mm^−1^, a total of 67,724 (θ_max_ = 27.58°), 8648 unique (*R*_int_ = 0.0541), 6093 [*I* > 2σ(*I*)], 837 parameters. GooF = 1.02, *R*_1_ = 0.0399, *wR*_2_ = 0.0938 [*I* > 2σ(*I*)], *R*_1_ = 0.0680, *wR*_2_ = 0.1136 (all data), max/min diff. peak 0.280/−0.233 e Å^−3^.

Crystal data of 1*2CH_3_CN: C_32_F_32_S_4_+2(CH_3_CN), M = 1202.67, triclinic, space group *P*-1, *a* = 10.1087(6), *b* = 10.1752(6), *c* = 20.9515(10) Å, α = 82.318(2), β = 81.616(2), γ = 82.275(3)°, *V* = 2098.3(2) Å^3^, *Z* = 2, d_calc_ = 1.904 g·cm^−3^, μ = 0.403 mm^−1^, a total of 67,731 (θ_max_ = 28.01°), 10,126 unique (*R*_int_ = 0.0435), 7405 [*I* > 2σ(*I*)], 669 parameters. GooF = 1.04, *R*_1_ = 0.0550, *wR*_2_ = 0.1488 [*I* > 2σ(*I*)], *R*_1_ = 0.0784, *wR*_2_ = 0.1726 (all data), max/min diff. peak 0.810/−0.447 e·Å^−3^.

### 3.5. General Procedure for the Synthesis of Dioxadithiacalixarenes ***8***, ***9***

Solution of trimethylamine (1.20 g, 12 mmol) and resorcinol (orcinol monohydrate) (2 mmol) in 10 mL of dimethylformamide was added dropwise to a stirred solution of 1.50 g of a mixture of perfluorinated *m*- and *p*-xylenes (~4 mmol of the *meta-*isomer) in 40 mL of dimethylformamide. The solution was stirred for 3 h at 20 °C and 30 min at 90 °C, then 0.63 g (2 mmol) of dithioresorcinol **4** was introduced and the mixture was stirred for 3 h at 90 °C. The solvent was evaporated in vacuum, and by column chromatography on silica gel using CCl_4_ as eluent, the solid product was isolated. Pure dioxadithiacalixarenes **8, 9,** were obtained by crystallization of the solid product from CCl_4_.

#### 3.5.1. 11,17,23,25,26,27-Hexafluoro-10,12,16,18,22,24-hexakis(trifluoromethyl)-2,8-dioxa-14,20-dithiapentacyclo[19.3.1.1^3,7^.1^9,13^.1^15,19^]octacosa-1(25),3(28),4,6,9(27),10,12,15(26),16,18,21,23-dodecaene **8**:

White solid (1.17 g), containing according ^19^F NMR data dioxadithiacalixarene 8 and tetrathiacalixarene 1 (94:6). mp 225–231 °C. ^19^F NMR (CDCl_3_): δ −119.5 (dq, 2F, J_F(25)-F(23)_ = 14 Hz, J_F(25)-CF3(22)_ = 6 Hz, F-25,27), −112.8 (qqd, 2F, J_F(11)-CF3(12)_ = 35 Hz, J_F(11)-CF3(10)_ = 28 Hz, J_F(11)-F(27)_ = 14 Hz, F-11,23), −112.2 (septet d, 1F, J_F(17)-2CF3(16,18)_ = 36 Hz, J_F(17)-F(26)_ = 15 Hz, F-11,23), −96.6 (dm, 1F, J_F(26)-F(17)_ = 15 Hz, F-26), −58.0 (d, 6F, J_CF3(10)-F(11)_ = 28 Hz, CF_3_(10,24)), −55.3 (dd, 6F, J_CF3(12)-F(11)_ = 36 Hz, J_CF3(12)-F(27)_ = 6 Hz, CF_3_(12,22)), −55.1 (dd, 6F, J_CF3(16)-F(17)_ = 36 Hz, J_CF3(16)-F(26)_ = 4 Hz, CF_3_(16,18)). ^19^F NMR (OC(CD_3_)_2_): δ −118.5 (dq, 2F, F-25,27), −113.9 (qqd, 2F, F-11,23), −111.7 (septet d, 1F, F-11,23), −92.6 (dm, 1F, F-26), −55.6 (d, 6F, CF_3_(10,24)), −53.1 (dd, 6F, CF_3_(12,22)), −52.7 (dd, 6F, CF_3_(16,18)). ^1^H NMR (CDCl_3_): δ 5.45 (m, 1H, H-28), 7.17 (dd, 2H, *J* = 8.3 Hz, *J* = 2.2 Hz, H-4,6), 7.56 (t, 1H, *J* = 8.3 Hz, H-5). ^1^H NMR (OC(CD_3_)_2_): δ 6.25 (m, 1H, H-28), 7.14 (dd, 2H, H-4,6), 7.55 (t, 1H, H-5). Anal. Calcd for C_30_H_4_F_24_O_2_S_2_: C, 39.32; H, 0.44; F, 49.75; S, 7.00%; M 916. Found: C, 38.72; H, 0.41; F, 49.91; S, 6.89%; M 911. GC–MS, *m/z*: 458 (M^++^, M 916).

#### 3.5.2. 11,17,23,25,26,27-Hexafluoro-5-methyl-10,12,16,18,22,24-hexakis(trifluoromethyl)-2,8-dioxa- 14,20-dithiapentacyclo[19.3.1.1^3,7^.1^9,13^.1^15,19^]octacosa-1(25),3(28),4,6,9(27),10,12,15(26),16,18,21,23- dodecaene **9**:

White solid (1.02 g, 55%). mp 161–165 °C. ^19^F NMR (CDCl_3_): δ −119.5 (dq, 2F, J_F(25)-F(23)_ = 14 Hz, J_F(25)-CF3(22)_ = 6 Hz, F-25,27), −112.9 (qqd, 2F, J_F(11)-CF3(12)_ = 36 Hz, J_F(11)-CF3(10)_ = 28 Hz, J_F(11)-F(27)_ = 14 Hz, F-11,23), −112.3 (septet d, 1F, J_F(17)-2CF3(16,18)_ = 36 Hz, J_F(17)-F(26)_ = 15 Hz, F-11,23), −96.6 (dm, 1F, J_F(26)-F(17)_ = 15 Hz, F-26), −57.9 (d, 6F, J_CF3(10)-F(11)_ = 28 Hz, CF_3_(10,24)), −55.3 (dd, 6F, J_CF3(12)-F(11)_ = 36 Hz, J_CF3(12)-F(27)_ = 6 Hz, CF_3_(12,22)), −55.1 (dd, 6F, J_CF3(16)-F(17)_ = 36 Hz, J_CF3(16)-F(26)_ = 4 Hz, CF_3_(16,18)). ^19^F NMR (OC(CD_3_)_2_): δ −118.4 (dq, 2F, F-25,27), −113.9 (qqd, 2F, F-11,23), −111.6 (septet d, 1F, F-11,23), −92.6 (dm, 1F, F-26), −55.6 (d, 6F, CF_3_(10,24)), −53.1 (dd, 6F, CF_3_(12,22)), −52.7 (dd, 6F, CF_3_(16,18)). ^1^H NMR (CDCl_3_): δ 2.38 (s, 3H, CH_3_), 5.08 (m, 1H, H-28), 6.82 (d, 2H, J_H(4,6)-H(28)_ = 4 Hz, H-4,6). ^1^H NMR (OC(CD_3_)_2_): δ 2.40 (s, 3H, CH_3_), 6.00 (m, 1H, H-28), 6.95 (d, 2H, H-4,6). ^13^C NMR (OC(CD_3_)_2_): δ 21.2 (C(5)-CH_3_), 97.2 (C-28), 114.1 (C-4,6), 116.3 (qd, ^2^J_CF_ = 33.0 Hz, ^2^J_CF_ = 14.9 Hz, C-CF_3_), 116.5 (qd, ^2^J_CF_ = 32.5 Hz, ^2^J_CF_ = 13.2 Hz, C-CF_3_), 121.9 (qd, ^2^J_CF_ = 32.4 Hz, ^2^J_CF_ = 13.9 Hz, C–CF_3_), 122.0 (q, ^1^J_CF_ = 274.9 Hz, CF_3_), 122.6 (q, ^1^J_CF_ = 276.4 Hz, CF_3_), 122.8 (q, ^1^J_CF_ = 275.8 Hz, CF_3_), 127.3 (d, ^2^J_CF_ = 24.9 Hz, C–S), 128.4 (d, ^2^J_CF_ = 17.4 Hz, C–S), 144.2 (C-5), 144.9 (dd, ^2^J_CF_ = 16.7 Hz, J_CF_ = 5.0 Hz, C(1,9)-0), 152.4 (dd, ^1^J_CF_ = 249.8 Hz, J_CF_ = 3.7 Hz, C–F), 155.1 (d, ^1^J_CF_ = 267.8 Hz, C–F), 155.4 (d, ^1^J_CF_ = 271.8 Hz, C–F), 158.4 (d, ^1^J_CF_ = 246.9 Hz, C–F), 159.1 (C(3,7)-0). Anal. Calcd for C_31_H_6_F_24_O_2_S_2_: C, 40.02; H, 0.65; F, 49.00; S, 6.89%; M 930. Found: C, 40.30; H, 0.98; F, 48.91; S, 6.88%; M 926.

### 3.6. Synthesis of Tetraoxadithiacalixarene ***12***

Thiourea (0.76 g, 10 mmol) was added to a stirred solution of 1.27 g (1.9 mmol) of 1,3-bis(2,4-bis(trifluoromethyl)trifluorophenoxy)benzene **6** in 50 mL of dimethylformamide. The solution was stirred for 15 h at 90 °C, and the solvent was evaporated in vacuum. By column chromatography on silica gel using CCl_4_ as eluent, 1.32 g of the viscous material containing (GC–MS) 91% tetraoxadithiacalixarene **12** and 5% dioxadithiacalixarene **8** was isolated. Double-play crystallization of viscous material from CCl_4_ gave 0.76 g of tetraoxadithiacalixarene 12*CCl_4_.

11,17,29,35,37,38,40,41-Octafluoro-10,12,16,18,28,30,34,36-octakis(trifluoromethyl)-2,8,20,26- tetraoxa-14,32-dithiaheptacyclo[31.3.1.1^3,7^.1^9,13^.1^15,19^.1^21,25^.1^27,31^]dotetraconta-1(37),3(42),4,6,9(41),10,12,15 (40),16,18,21(39),22,24,27(38),28,30,33,35-octadecaene **12**:

White solid (0.76 g, 47%). mp 291 °C (decomp.). ^19^F NMR (OC(CD_3_)_2_): δ −117.0 (m, 4F, F-37,38,40,41), −112.4 (m, 4F, F-11,17,29,35), −55.8 (d, 12F, J_CF3(10)-F(11)_ = 26 Hz, CF_3_(10,18,28,36)), −53.1 (d, 12F, J_CF3(12)-F(11)_ = 34 Hz, CF_3_(12,16,30,34)). ^1^H NMR (OC(CD_3_)_2_): δ 6.14 (m, 2H, H-39,42), 6.85 (dd, 4H, J_H(4)-H(5)_ = 8.4 Hz, J_H(4)-H(42)_ = 2.3 Hz, H-4,6,22,24), 7.38 (t, 2H, J_H(5)-H(4,6)_ = 8.4 Hz, H-5,23). ^13^C NMR (OC(CD_3_)_2_): δ 102.7 (C-39,42), 111.8 (C-4,6,22,24), 116.5 (qd, ^2^J_CF_ = 33.4 Hz, ^2^J_CF_ = 15.6 Hz, C–CF_3_), 118.3 (qd, ^2^J_CF_ = 32.7 Hz, ^2^J_CF_ = 13.2 Hz, C–CF_3_), 121.8 (q, ^1^J_CF_ = 275.0 Hz, CF_3_), 122.8 (q, ^1^J_CF_ = 276.4 Hz, CF_3_), 128,7 (d, ^2^J_CF_ = 18.5 Hz, C–S), 132.5 (C-5,23), 144.3 (dd, ^2^J_CF_ = 17.8 Hz, J_CF_ = 4.8 Hz, C(1,9,19,27)-0), 152.8 (d, ^1^J_CF_ = 249.1 Hz, C–F), 155.6 (d, ^1^J_CF_ = 268.9 Hz, C–F), 158.7 (C(3,7,21,25)-0). Anal. Calcd for C_44_H_8_F_32_O_4_S_2_*CCl_4_: C, 37.89; H, 0.57; F, 42.62; Cl, 9.94; S, 4.50%; M 1272. Found: C, 37.80; H, 0.70; F, 42.68; Cl, 9.96; S, 4.79%; M 1272.

### 3.7. Synthesis of Tetraoxathiacalixarene ***15***

Thiourea (0.23 g, 3 mmol) was added to a stirred solution of 1.08 g (1.1 mmol) of pentaphenyl ether **13** in 50 mL of dimethylformamide. The solution was stirred for 15 h at 90 °C, and the solvent was evaporated in vacuum. By column chromatography on silica gel using CCl_4_ as eluent, 0.99 g of the viscous material containing (GC–MS) 61% tetraoxathiacalixarene **15**, 20% pentaphenylether **13**, and 17% dimethylformamide was isolated. Double-play crystallization of viscous material from CCl_4_ gave 0.36 g of tetraoxathiacalix[5]arene **15**.

11,23,29,31,32,34-Hexafluoro-10,12,22,24,28,30-hexakis(trifluoromethyl)-2,8,14,20-tetraoxa-26- thiahexacyclo[25.3.1.1^3,7^.1^9,13^.1^15,19^.1^21,25^]pentatriaconta-1(31),3(35),4,6,9(34),10,12,15(33),16,18,21(32),22,24,27,29-pentadecaene **15**:

White solid (0.36 g, 33%). mp 194 ^°^C (decomp.). ^19^F NMR (OC(CD_3_)_2_): δ −134.2 (d, 1F, J_F(34)-F(11)_ = 12 Hz, F-34), −116.4 (septet d, 1F, J_F(11)-CF3(10,12)_ = 29 Hz, J_F(11)-F(34)_ = 12 Hz, F-11), −116.0 (d, 2F, J_F(31)-F(29)_ = 10 Hz, F-31,32), −113.3 (unresolved m, 2F, F-23,29), −55.9 (d, 6F, J = 27 Hz, 2CF_3_), −55.0 (d, 6F, J = 29 Hz, 2CF_3_), −53.2 (d, 6F, *J* = 30 Hz, 2CF_3_). ^1^H NMR (OC(CD_3_)_2_): δ 6.92 (m, 1H, H-33,35), 6.94 (dd, 2H, J_H(4)-H(5)_ = 8.4 Hz, J_H(4)-H(35)_ = 1.4 Hz, H-4,18), 7.01 (dd, 2H, J_H(6)-H(5)_ = 8.1 Hz, J_H(6)-H(35)_ = 1.5 Hz, H-6,16), 7.38 (dd, 2H, *J* = 8.4 Hz, *J* = 8.1 Hz, H-5,17). ^13^C NMR (OC(CD_3_)_2_): δ 105.7 (C-33,35), 109.0 (qd, ^2^J_CF_ = 33.1 Hz, ^2^J_CF_ = 14.5 Hz, C–CF_3_), 112.9 (C-4,18), 113.4 (C-6,16), 116.5 (qd, ^2^J_CF_ = 33.1 Hz, ^2^J_CF_ = 15.4 Hz, C–CF_3_), 118.1 (qd, ^2^J_CF_ = 33.1 Hz, ^2^J_CF_ = 13.0 Hz, C–CF_3_), 121.7 (q, ^1^J_CF_ = 274.4 Hz, CF_3_), 121.8 (q, ^1^J_CF_ = 274.0 Hz, CF_3_), 122.0 (q, ^1^J_CF_ = 276.0 Hz, CF_3_), 126,7 (d, ^2^J_CF_ = 20.9 Hz, C–S), 131.3 (C-5,17), 143.7 (dd, ^1^J_CF_ = 254.9 Hz, J_CF_ = 4.2 Hz, C–F), 144,6 (d, ^2^J_CF_ = 16.3 Hz, C–O), 144,7 (d, ^2^J_CF_ = 18.0 Hz, C–O), 153.1 (dd, ^1^J_CF_ = 251.3.1 Hz, J_CF_ = 2.2 Hz, C–F), 154.3 (d, ^1^J_CF_ = 264.8 Hz, C–F), 154.7 (d, ^1^J_CF_ = 267.9 Hz, C–F),157.7 (C-0), 158.4 (C-0). Anal. Calcd for C_36_H_8_F_24_O_4_S: C, 43.57; H, 0.81; F, 45.94; S, 3.23%; M 992. Found: C, 43.30; H, 0.99; F, 45.82; S, 3.30%; M 994.

## 4. Conclusions

Thus, it has been shown that perfluoro-m-xylene and 2,5-difluoro-4,6-bis (trifluoromethyl)benzene-1,3-dithiol are convenient building blocks for the synthesis of polyfluorinated thiacalixarenes. Sequential interaction of perfluoro-m-xylene with resorcinols and 2,5-difluoro-4,6-bis (trifluoromethyl)benzene-1,3-dithiol or thiourea leads to the formation of polyfluorinated oxathiacalixarenes containing 4–6 aromatic nuclei in the macrocycle. Based on the analysis of X-ray diffraction data and ^1^H and ^19^F NMR spectra, it has been shown that polyfluorinated tetratia- and dioxadithiacalix[4]arenes are in the 1,3-alternate conformation in the crystal and in solution.

## Data Availability

The data presented in this study are available on request from the corresponding author.

## Supplementary Materials

The following are available, copy ^1^H, ^19^F, ^13^C NMR spectral data of compounds **1, 9, 12, 13** and **15** (file type pdf).

## Supplementary X-Ray Crystallographic Data

>CCDC 2051376 (**1**), 2051377 (**1*2CH_3_CN**) contain the supplementary crystallographic data for this paper. These data can be obtained free of charge from the Cambridge Crystallographic Data Centre via https://www.ccdc.cam.ac.uk/structures (or from e-mail: deposit@ccdc.cam.ac.uk).

## Sample Availability

Samples of the compounds **1, 8, 9, 12** are available from the authors.
