# Supplementary material for: Synthesis of Polyfluorinated Thia- and Oxathiacalixarenes Based on Perfluoro-m-xylene
Source: Molecules. 2021 Jan 20;26(3):526. doi: 10.3390/molecules26030526 (PMC7864041; doi:10.3390/molecules26030526)
Supplement: Supplementary file 1 [file molecules-26-00526-s001.zip › Figure S11_H.pdf]

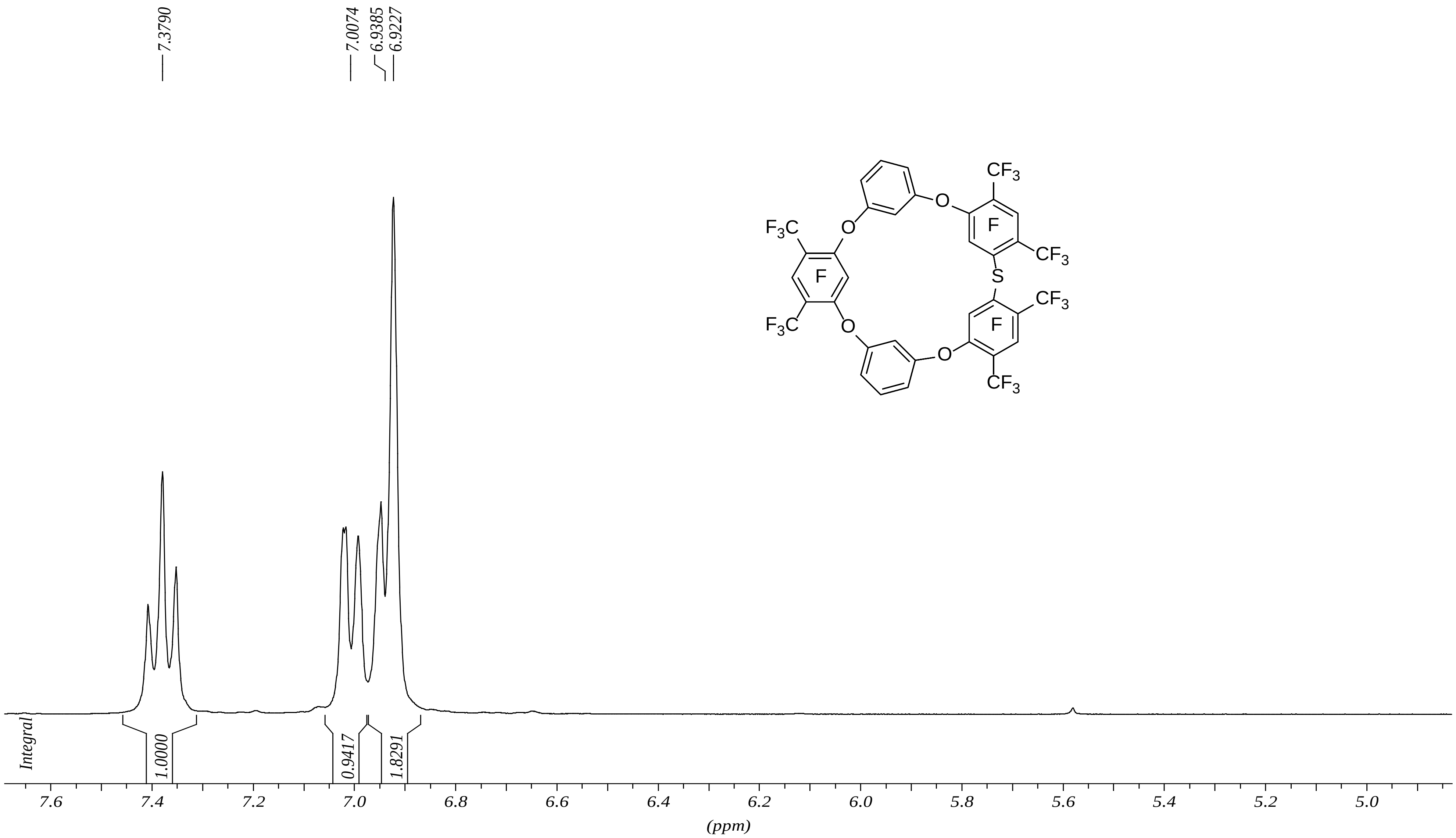

Figure S11. ^{1}H NMR spectra (acetone-d6) 11,23,29,31,32,34-hexafluoro-10,12,22,24,28,30-hexakis(trifluoromethyl)-2,8,14,20-tetraoxa-26-thiahexacyclo[25.3.1.$1^{3,7}$.$1^{9,13}$.$1^{15,19}$.$1^{21,25}$]pentatriaconta-1(31),3(35),4,6,9(34),10,12,15(33),16,18,21(32),22,24,27,29-pentadecaene **15.**
