# Supplementary material for: Synthesis of Polyfluorinated Thia- and Oxathiacalixarenes Based on Perfluoro-m-xylene
Source: Molecules. 2021 Jan 20;26(3):526. doi: 10.3390/molecules26030526 (PMC7864041; doi:10.3390/molecules26030526)
Supplement: Supplementary file 1 [file molecules-26-00526-s001.zip › Figure S2_H.pdf]

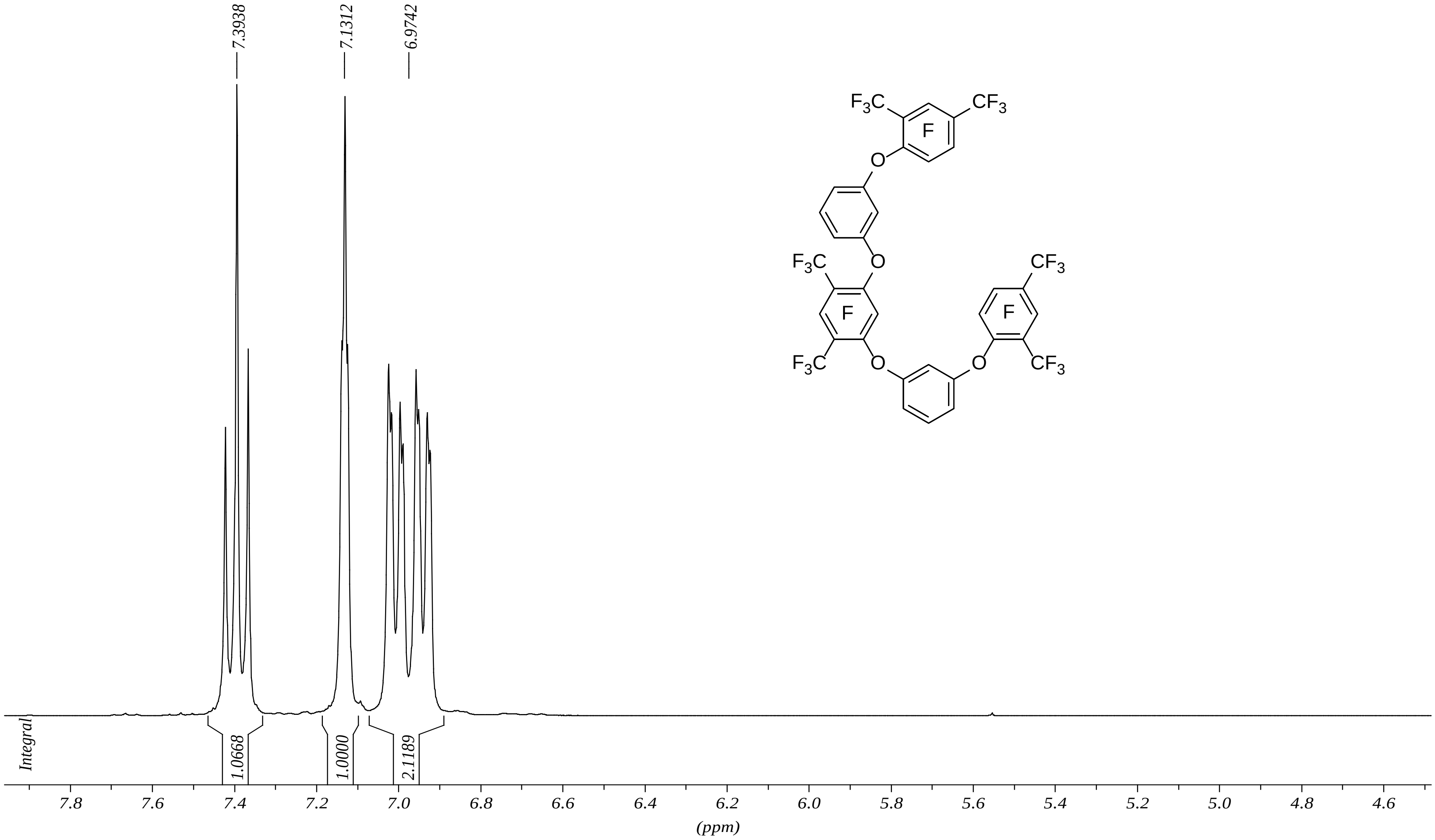

Figure S2. ^{1}H NMR spectra (acetone-d6) of 1,3-bis{3-[3,5,6-trifluoro-2,4-bis(trifluoromethyl)phenoxy]phenoxy}-2,5-difluoro-4,6-bis(trifluoro methyl)benzene **13.**
