# Supplementary material for: Synthesis of Polyfluorinated Thia- and Oxathiacalixarenes Based on Perfluoro-m-xylene
Source: Molecules. 2021 Jan 20;26(3):526. doi: 10.3390/molecules26030526 (PMC7864041; doi:10.3390/molecules26030526)
Supplement: Supplementary file 1 [file molecules-26-00526-s001.zip › Figure S3_F.pdf]

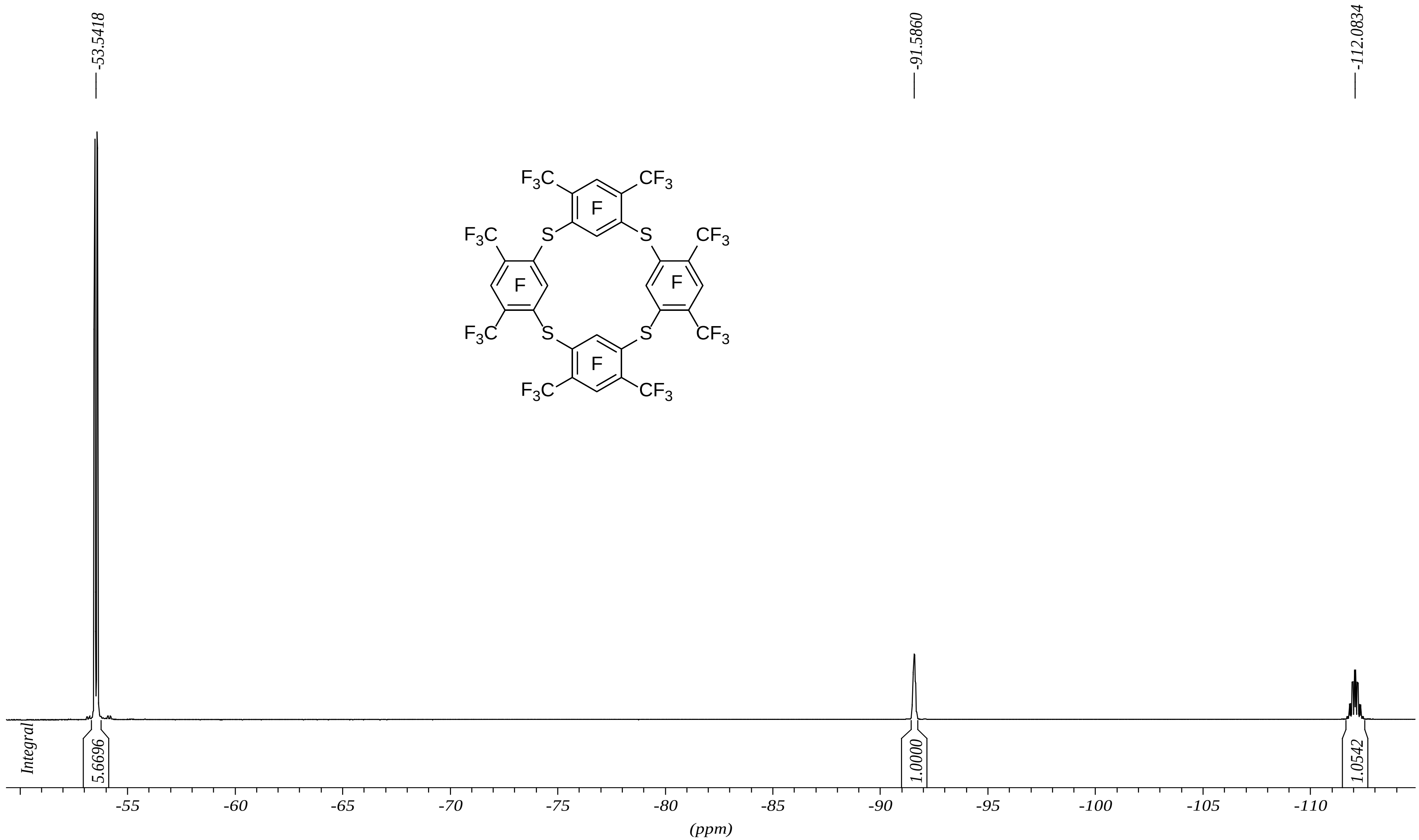

Figure S3. ^{19}F NMR spectra (acetone-d6) of 5,11,17,23,25,26,27,28-octafluoro-4,6,10,12,16,18,22,24-octakis(trifluoromethyl)-2,8,14,20-tetrathia-pentacyclo[19.3.1.$1^{3,7}$.$1^{9,13}$.$1^{15,19}$]octacosa-1(25),3(28),4,6,9(27),10,12,15(26),16,18,21,23-dodecaene **1.**
