# Supplementary material for: Synthesis of Polyfluorinated Thia- and Oxathiacalixarenes Based on Perfluoro-m-xylene
Source: Molecules. 2021 Jan 20;26(3):526. doi: 10.3390/molecules26030526 (PMC7864041; doi:10.3390/molecules26030526)
Supplement: Supplementary file 1 [file molecules-26-00526-s001.zip › Figure S8_H.pdf]

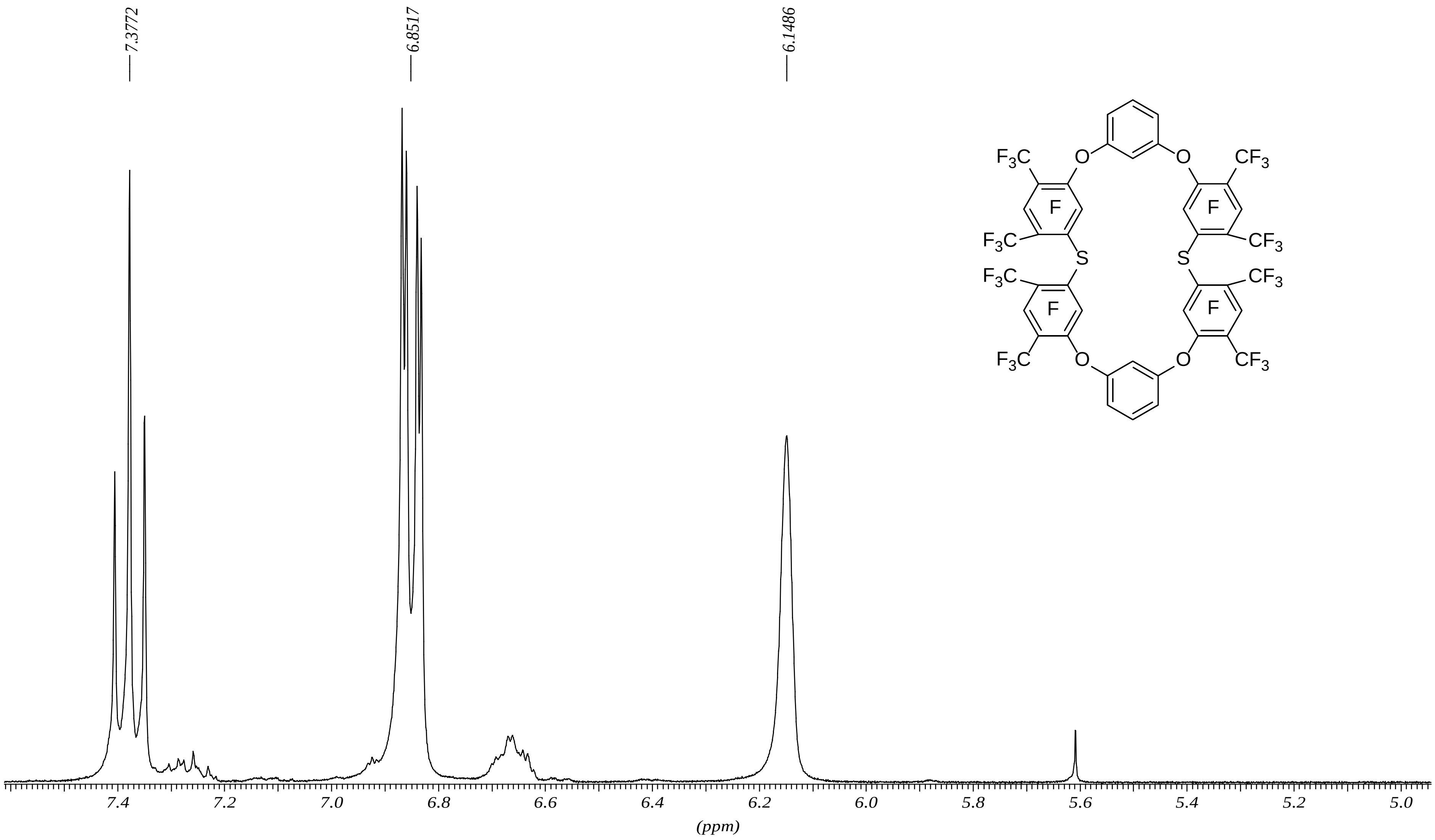

Figure S8. ^{1}H NMR spectra (acetone-d6) 11,17,29,35,37,38,40,41-octafluoro-10,12,16,18,28,30,34,36-octakis(trifluoromethyl)-2,8,20,26- tetraoxa-14,32-dithiaheptacyclo[31.3.1.1^{3,7}.1^{9,13}.1^{15,19}.1^{21,25}.1^{27,31}]dotetraconta-1(37),3(42),4,6,9(41),10,12,15 (40),16,18,21(39),22,24,27(38),28,30,33,35-octadecaene **12.**
